# Can Self-Regulatory Strength Training Counter Prior Mental Exertion? A Systematic Review of Randomized Controlled Trials

**DOI:** 10.3389/fpubh.2022.904374

**Published:** 2022-06-10

**Authors:** He Sun, Kim Geok Soh, Mohd Rozilee Wazir Norjali Wazir, Cong Ding, Tingting Xu, Dong Zhang

**Affiliations:** ^1^Department of Sport Studies, Faculty of Education Studies, Universiti Putra Malaysia, Selangor, Malaysia; ^2^School of Journalism and Communication, Zhengzhou University, Zhengzhou, China; ^3^School of Physical Education and Sports, SooChow University, Suzhou, China

**Keywords:** self-regulation, ego depletion, mental fatigue, self-regulatory strength, physical performance, cognitive performance

## Abstract

**Background:**

Prior mental exertion consumes self-regulation and influences any subsequent physical or cognitive performance according to the strength model of self-regulation. However, the counteractive effect of self-regulatory strength training remains unclear.

**Objective:**

This study aims to report a comprehensive systematic review investigating self-regulatory strength training programmes on physical or cognitive performance.

**Methods:**

To select relevant studies from the available literature, a thorough search was conducted on PubMed, Web of Science, EBSCOhost (CENTRAL, Psychology and Behavioral Sciences Collection, SPORTDicus), Scopus, and Google Scholar, as well as the sources of reference for gray literature. Only randomized controlled trials involving healthy humans, strength-based self-regulation training programmes with comparable protocols, and a physical or cognitive task associated with the study were selected for the current review. The Grading of Recommendations Assessment Development and Evaluation (GRADE) framework was used to develop the summary of findings.

**Results:**

Twelve articles were included based on the selection criteria. Evidence certainty for outcomes was graded as either low or very low level. The majority of the studies reported that self-regulatory strength training programmes can significantly counter prior mental exertion and decrement of performance, while only one study did not find such improvement. According to the strength model, a period of training increased the ‘self-regulatory muscle.'

**Conclusion:**

*Strength* is an important ingredient in the resource model of self-regulation and can be trained to counter prior mental exertion and improve subsequent physical and cognitive performance. The training effects are cross-domain (e.g., emotional and cognitive domains; higher and lower levels of executive functions). However, motivation plays a key role to mobilize this resource. Future studies should examine the mechanism that underlies the *strength*.

**Systematic Review Registration:**

https://inplasy.com/inplasy-2022-1-0060/, identifier: INPLASY202210060.

## Introduction

Self-regulation is the ability to adjust one's mental and physiological state adaptively to a given context, and it includes emotional, cognitive, behavioral, and physiological adaptation (1, 2). Humans must regularly exercise mental exertion and seize control over themselves to achieve the best performance. For example, to attain exemplary scores in school, a student must concentrate in class and exercise mental exertion to combat any internal (task-induced boredom) or external detractors of accomplishing goal attainment. Similarly, a cyclist must perform mental exertion to resist the urge to slow down despite suffering from body ache, or a soccer player must extract and interpret useful information while blocking out distractions from the complex competitive environment they are in for a prolonged period. Over the last two decades, there has been a growing body of evidence indicating that mental exertion has a long-term effect on one's physical and cognitive performance (3–6). Specifically, Englert and Wolff (5) demonstrated that depleted participants with low self-regulatory strength invested less effort to do the cycling test compared with those having increased self-regulatory strength, which is measured as a lower heart rate. Besides the physical performance, Furley et al. (6) found that depleted participants could not focus their attention on the task to make good decisions and block out additional irrelevant stimuli from the audition.

In recent years, several efforts have been made to synthesize the literature both narratively (7, 8) and quantitatively (9, 10), whereby performing mental exertion has led to a subsequent decrease in physical and cognitive performance across a wide range of tasks. However, these existing reviews mainly focus on the carryover effects, without exploring potential counteractive strategies. Finding effective interventions should be the next goal of studies in this field, which means that they should not be limited to simply demonstrating these negative effects. Therefore, a comprehensive synthesis and analysis of intervention methods are necessary.

The strength model of self-regulation (11, 12) has been utilized to describe performance decrements caused by previous mental exertion in the last two decades. *Strength*, also called energy, is required and can be depleted temporarily when individuals regulate the self (13, 14). The ability to conduct mental exertion is based on strength or this depletable resource as per the paradigm (9, 15). This depletable state is known as “ego depletion,” and it is thought to impair physical and cognitive performance. The “global” nature of such resources refers to the fact that all of the self-regulatory activities consume the same resource pool; for example, regulating an emotional or physiological response will affect performance in completely unrelated self-regulation demanding handgrip tasks (12). The model was well tested in a meta-analysis conducted by Hagger et al. (16) in 83 experiments with 10, 500 participants. Notably, the model indicates that the strength of self-regulation is similar to a “muscle” and can be exercised (17, 18), providing a way to minimize the negative effect of prior mental exertion and improve the subsequent performance, including physical or cognitive aspects. Muraven et al. (19) provided the first evidence that 2 weeks of training in self-regulatory strength (e.g., posture and mood regulation) can significantly reduce the susceptibility of fatigue induced by prior mental exertion and improve handgrip.

Fatigue is a sign of a decrease in available energy usage for future self-regulation, resulting in an inability to maintain current effort (20, 21). Thus, it is not surprising that the condition of mental fatigue is also induced by prior mental exertion, which has been reported several years ago [e.g., (22, 23)]. Many studies have shown that mental fatigue negatively influences a variety of physical and cognitive performance, such as cycling performance (24, 25), goal-directed attention (26), and inhibition (27). Furthermore, most current literature on mental fatigue focuses on sports performance. The negative effect has been corroborated in intermittent endurance (28–30), technical performance (31–33), and decision-making skills (34, 35).

On the other hand, individuals with a better capacity for self-regulation can be less vulnerable to mental fatigue and perform better in subsequent endurance tests (36). Additionally, Martin and colleagues (37) found professional cyclists showed greater resistance to mental fatigue because they must do routine training and follow a certain programme, and even restrict their diet, which could significantly strengthen their self-regulatory capacity. However, several studies indicated that the intervention to counter mental fatigue and improve the subsequent performance is still misty (35, 38–41). Also, because of the similarities in potential mechanism (decreased activation in areas that include the anterior cingulate cortex and prefrontal cortex) and methodology (e.g., dual-task paradigm) of investigations in two academic areas (ego depletion and mental fatigue), mounting studies have been merging them theoretically (42) and practically (10, 43). Thus, this review summarizes interventions in two study areas together, which provides deeper insights and suggests available interventions for prior mental exertion.

As a result, the review develops a comprehensive evaluation of the intervention's enhanced self-regulation strength, providing evidence for future research to explore particular techniques to counteract earlier mental exertion. Particularly, only the studies that investigated the outcome of physical (the measurement of the capacity to carry out any tasks related to the action) and/or cognitive performance (the measurement of cognitive abilities such as inhibition, decision-making, problem-solving, etc.) are selected.

## Methodology

**Figure 1:**
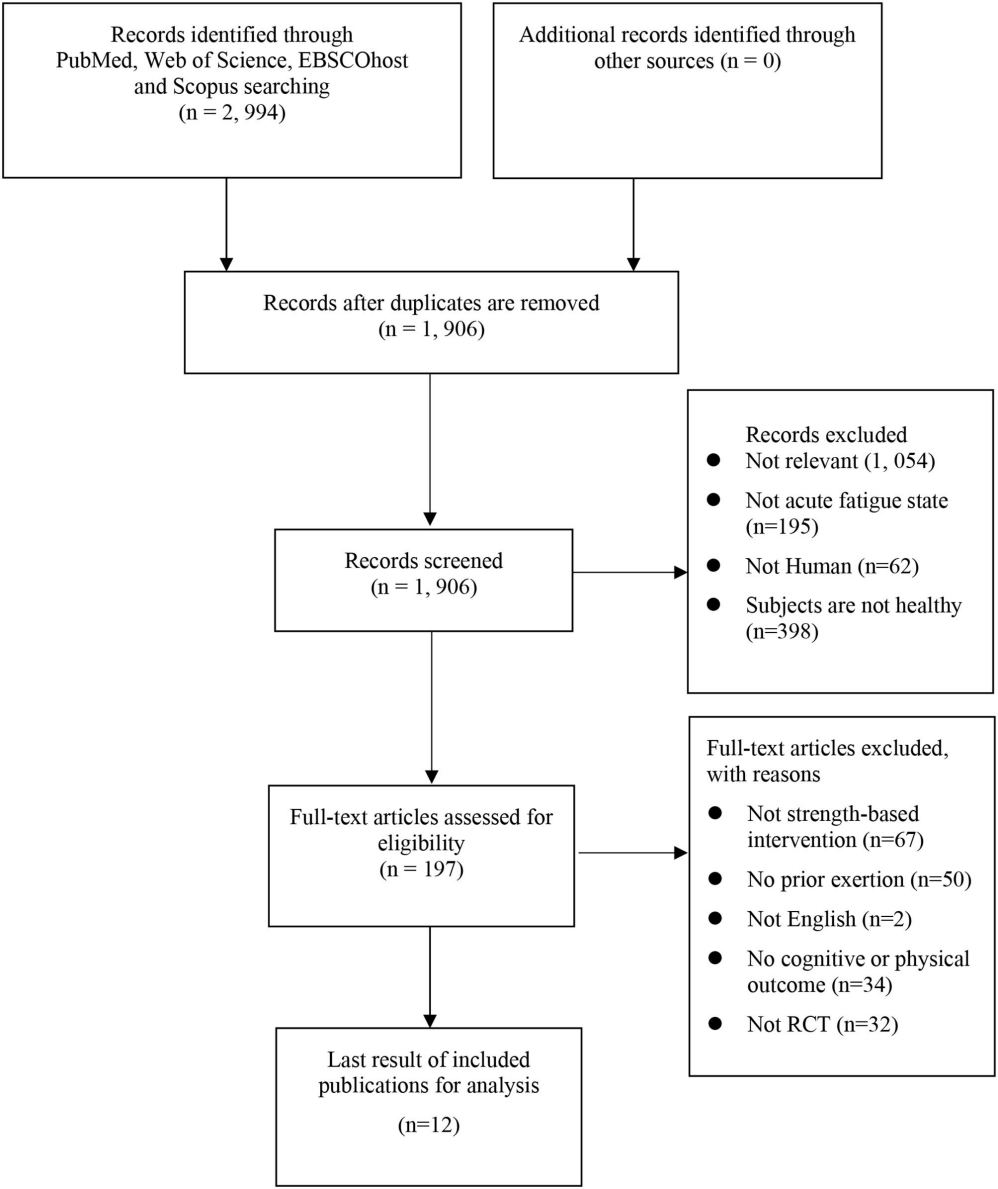
PRISMA summary of the selection procedure.

This review's reporting adheres to the preferred reporting items checklist used in the systematic (PRISMA) protocol (44). A systematic literature search was carried out using four main databases, namely, PubMed, Web of Science, EBSCOhost (CENTRAL, Psychology and Behavioral Sciences Collection, and SPORTDicus), and Scopus, for published works from 1999 onwards, which is the first publication year of self-regulatory strength training study (19), to January 2022 (Supplementary Table S1). EBSCOhost comprises numerous sub-databases; however, only Cochrane Central Register of Controlled Trials (CENTRAL), Psychology and Behavioral Science Collection, and SPORTDicus were selected, due to the relevance of their content. In addition, citations and reference lists were searched to identify any additional studies. The details of the search results are presented in Figure 1. Data searching was assisted by experienced librarians, who ensured the reliability of the searching method.

### Eligibility Criteria

The PICOS method was utilized to identify the literature (Table 1), implying that studies were eligible for the review if they conformed to the following criteria: (a) evaluated self-regulation training in healthy humans; (b) included physical or cognitive performance outcomes after participating in a mental exertion task; (c) reported a randomized controlled trial; (d) peer-reviewed literature published in English; and (e) included records published during the period from 1999 to 2022.

**Table 1:** PICOS (participation, intervention, comparison, outcome, and study design).

**PICOS**	**Criteria**
Participation	Healthy human
Intervention	Strength based self-regulatory training programme
Comparison	Intervention vs. No intervention group
Outcome	Cognitive or physical performance
Study design	Randomized controlled trial

*Strength* is defined as the energy that is required and can be depleted temporarily when individuals regulate the self (13). The training programmes that were developed and aimed to increase the energy of regulating self were included. Moreover, only the studies that investigated the outcome of physical (the measurement of the capacity to carry out any tasks related to the action) and/or cognitive performance (the measurement of cognitive abilities such as inhibition, decision-making, problem-solving, etc.) were selected.

When searching the databases, the following keywords, truncation, and Boolean operators were employed separately and in combination (Supplementary Table S1). The search was centered on the sources of reference in the identified reviews for additional literature, which would not have surfaced in the search results if only the primary databases were utilized.

Two reviewers separately examined the abstracts and titles of studies from other sources, and the results of publications acquired using the search strategy to identify research that may fit the inclusion criteria mentioned earlier. After screening, 197 papers were selected for a full-text review. Two reviewers independently reviewed these papers for inclusion. A third reviewer was consulted to resolve any discrepancies.

### Protocol and Registration

The protocol of methods and planned analyses applied in this systematic review was registered in INPLASY (ref. INPLASY202210060). There are protocols in INPLASY that examine the counteractive effects intervention has on prior mental exertion (e.g., mental fatigue), such as supplements, but none of the studies focus on self-regulatory strength training toward physical and/or cognitive performance concurrently. As such, the novelty of the proposed protocol was assured.

### Risk of Bias and the Certainty of Evidence Assessment

The Revised Cochrane Risk of Bias tool for randomized trials (RoB 2.0) was used to assess the risk of bias in the individual studies. Each of these five categories earned a rating of “low risk of bias,” “high risk of bias,” or “some concerns of bias” according to the signaling questions specified in the RoB 2.0 tool. Lastly, the overall risk of biased judgement was formed for each study. The guidelines provided by the Cochrane community were followed by reviewers (S.H. and X.T.).

Given the heterogeneity across measurement and training programmes, the pooling of data for a meta-analysis was not done. Therefore, only narrative synthesis of findings with outcomes was developed and presented in the summary of findings table (Table 2). Grading of Recommendations, Assessment, Development, and Evaluations (GRADE) with the “GRADEpro” online tool was recruited to facilitate the synthesis and check the certainty (quality) of evidence regarding limitations of studies (e.g., risk of bias, inconsistency of training programme effects, indirectness, imprecision, or other factors) (55–57). The certainty of evidence assessment was done by two reviewers (S.H. and X.T.). The results for the certainty of evidence assessment and risk of bias were verified by the review team, which has systematic review methodology experts (S. K. G. and Z. Z). Any disagreements were resolved by further discussion in the team.

**Table 2:** Overview of the included publications details.

**NO**.	**References**	**Population characteristics**	**Intervention**	**Type of training**	**Prior mental exertion**	**Duration of the prior mental exertion**	**Outcome**	**Domain of the outcome**	**Similarity**
1	Muraven et al. (19)	69 undergraduates Sex: 42 ♂; 27 ♀ (Exp: 31 vs. Con: 38)	Length: 2 weeks.	Posture regulation Mood regulation	Thought-suppression task	5 min	Posture regulation group: Handgrip task: Drop in the task↓ Mood regulation group Handgrip task: Drop in the task ↔	Physical domain	M
2	Oaten and Cheng (45)	45 undergraduates Sex: 7♂; 38♀ (Exp: 28 vs. Con 17)	Length: 8 weeks	Academic study program	Thought-suppression task	5 min	Visual tracking task: Error↓in the exam period Self-efficacy ↔ Perceived Stress ↔ Emotional distress ↔	Inhibition	M
3	Oaten and Cheng (46)	24 sedentary undergraduates Sex: 6 ♂; 18♀ Age: 24 ± 6 (Exp: 24 vs. Con: 24)	Length: 4 weeks; Freq: 3–4 times/week	Cardiovascular exercise	Thought-suppression task	5 min	Visual tracking task: Error↓ Self-efficacy ↔ Perceived stress ↔	Inhibition	U
4	Gailliot et al. (1) Study 1	38 undergraduates Sex: 24♂; 14♀ (Exp: 19 vs. Con: 19)	Length: 2 weeks	Modifying verbal mannerism	Stereotype-suppression task	UA	Anagram task: Number↑in low-motivation group;	Problem-solving	U
	Study 2	98 undergraduates Sex: 31♂; 67♀ (Exp: 45 vs. Con: 53)	Length: 2 weeks	Exp 1: Verbal mannerism modifying Exp 2: Non-dominant hand using	Stereotype-suppression task	UA	Anagram task: Number↑in low-motivation Number ↔ in high-motivation Effort ↑in low-motivation Mood ↔ Arousal ↔	Problem-solving	U
	Study 4	52 undergraduates Sex: 11♂; 41♀ (Exp: 26 vs. Con: 26)	Length: 2 weeks	Non-dominant hand using	Stereotype-suppression task	UA	Stroop task: Accuracy↑ Reaction time ↔	Inhibition	M
5	Oaten and Cheng (47)	49 undergraduates Sex: 12 ♂; 37 ♀ (Exp: 29 vs. Con: 20)	Length: 4 months	Financial monitoring	Thought-suppression task	5 min	Visual tracking task: Error↓ Self-efficacy ↔ Perceived stress ↔ Emotional distress ↔	Inhibition	M
6	Denson et al. (48)	70 undergraduates Sex: 16 ♂; 54 ♀ Age: 20.30 ± 2.99 (Exp: 35 vs. Con: 35)	Length: 2 weeks	Non-dominant hand using	Anger induction	12 min	Taylor Aggression Paradigm: Aggressive behavior↓	Inhibition	M
7	Cranwell et al. (49) Study 1	29 university students and staff Sex: 29♀ (Exp: 15 vs. Con: 14)	Length: 4 weeks; Freq: 3 times/day; Duration: 10 min	Stroop task	Stroop task	10 min	Stroop task: Reaction time↓ Handgrip task: Persistence duration↑	Inhibition	M
	Study 2	33 university students and staff Sex: 33♀ (Exp: 17 vs. Con: 16)	Length: 4 weeks; Freq: 3 times/day; Duration: 10 min	Complex counting task	Complex counting task	UA	Handgrip task: Persistence duration↑	Physical domain	U
8	Bertrams and Schmeichel (50)	49 undergraduates Sex: 11♂; 38 ♀ Age: 22.49 ± 3.50 (Exp: 25 vs. Con: 24)	Length: 1 weeks	Regular logical reasoning	Letter typing task	5 min	Anagram task: Number↑ Follow up test (after 1 week of post-test): Number ↔	Problem solving	M
9	Bray et al. (51)	41 undergraduates Sex: 15♂; 26♀ Age: 18.66 ± 1.56 (Exp: 21 vs. Con: 20)	Length: 2 weeks; Freq: 2 time/day; Duration: as long as possible	Isometric handgrip exercise	Stroop task	5 min	Maximal cardiovascular exercise: Time to fail↑ RPE ↔	Physical domain	U
10	Allom and Mullan (52) Study 1	82 undergraduates Sex: 16♂; 66♀ Age: 20.43 ± 4.86 (Exp 1: 25 vs. Exp 2: 29 vs. Con 28)	Length: 10 days; Freq: 1 time/day	Stop-signal task: Exp 1: Food specific inhibition Exp 2: General inhibition	Letter typing task	5 min	Vulnerability to depletion↓ 20 number trails of Stroop task: Exp 1 vs. Con: Reaction time ↔ Accuracy ↔ Exp 2 vs. Con Reaction time ↔ Accuracy ↔	Inhibition	M
	Study 2	78 university students and staff Sex: 17♂; 61♀ Age: 22.91 ± 5.81 (Exp 1: 27 vs. Exp 2: 26 vs. Con 25)	Length: 10 days; Freq: 1 time/day	Exp 1: Food specific inhibition Exp 2: General inhibition	Letter typing task	5 min	Vulnerability to depletion↓ 50 number trails of Stroop task: Exp 1 vs. Con: Reaction time↓ Exp 2 vs. Con Reaction time↓ Follow-up test ↔	Inhibition	M
11	Miles et al. (53)	174 undergraduates and postgraduates Sex: 71♂; 103 ♀ (Exp 1: 45 vs. Exp 2: 44 vs. Active Con 45 vs. No-contract Con 40)	Length: 6 weeks; Freq: 5 days/week	Exp 1: Cognitive (Stroop and stop-single task) training; Exp 2: behavioral (non-dominant hand) training;	Four consecutive tasks	UA	Handgrip task: Persistence duration ↔	Physical domain	M
12	Filipas et al. (54)	20 untrained young adults Sex: 6♂; 14♀ Age: 27.6 ± 6.2 (Exp: 20 vs. Con: 10)	Length: 4 weeks; Freq: 3 times/week; Duration: 60 min	Cycle ergometer: incremental maximal ramp;	45-min cognitive battery; 40-min Stroop task; and 5-min flanker task	90 min	Cycling ergometer Total distance↑	Physical domain	M

*Freq, frequency; U, unmatched; M, matched; UA, unavailable; ↑ the value is significantly higher in the experimental group compared to the control group; ↓ the value is significantly lower in the experimental group compared to the control group; ↔ no significant differences between experimental and control groups. ♂, male; ♀, female*.

## Results

### Study Selection

The study search yielded 1,906 unique publications. After screening, 12 studies met all of the eligibility criteria. A forward search (assessing the citations of the included publications) and backward search (assessing the reference lists of the included publications), and searching in Google Scholar provided no additional studies. Two independent reviewers showed agreement about the result. Figure 1 illustrates the study selection procedure.

### Risk of Bias

**Figure 2:**
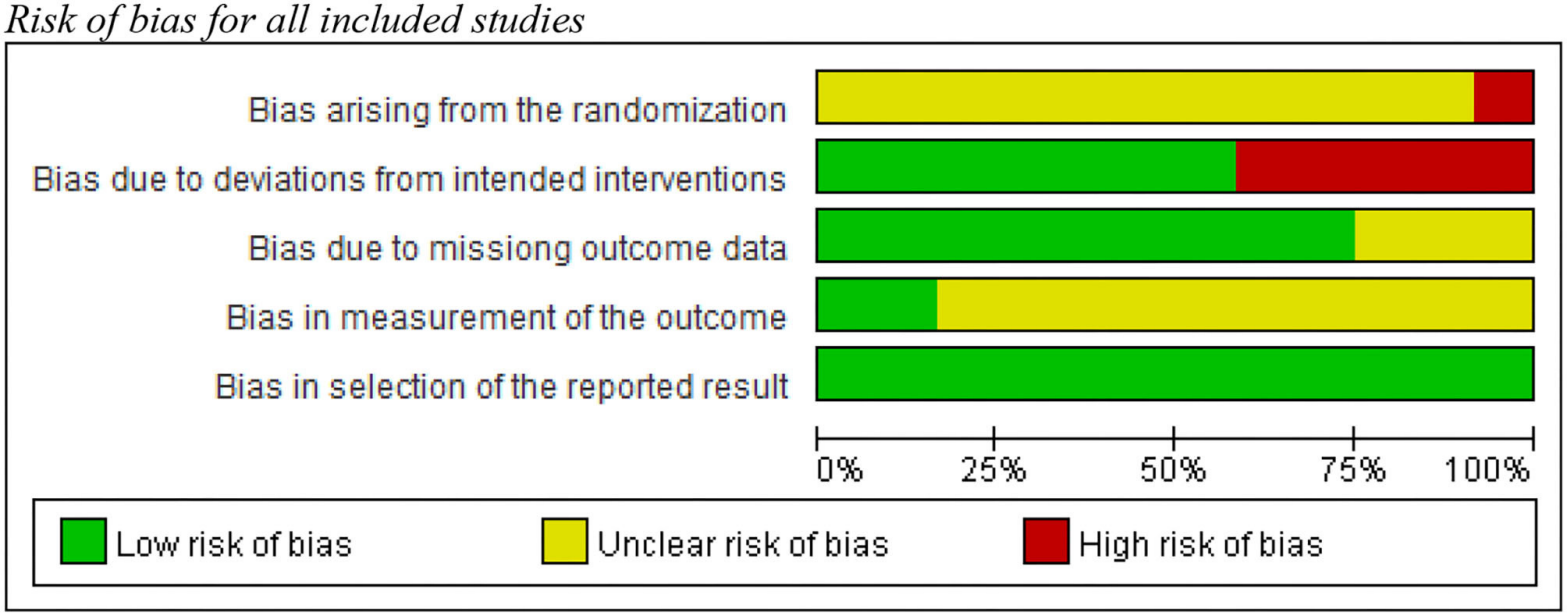
Risk of bias for all included studies.

**Figure 3:**
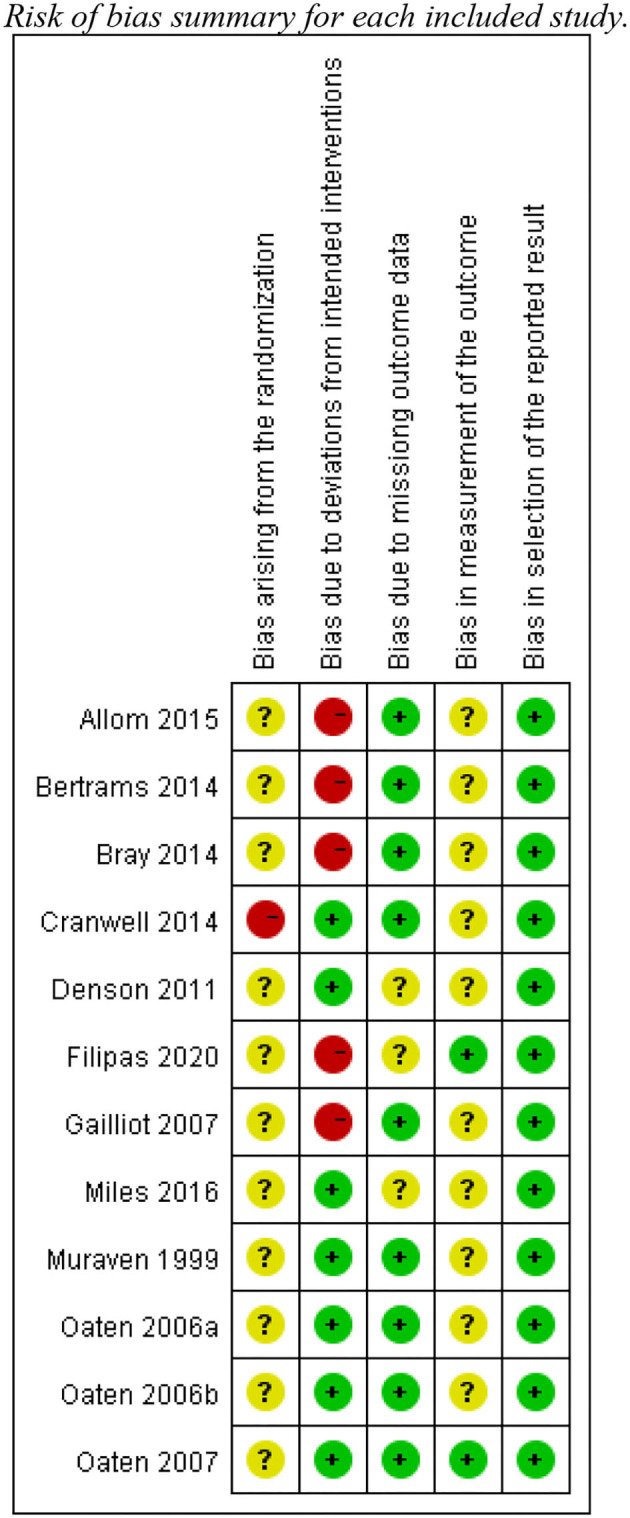
Risk of bias summary for each included study.

The risk bias of assessment for the 12 included studies with the RoB 2-tool showed that six studies had a high level of risk of bias, while the other studies scored low or unclear risk (Figures 2, 3). According to the signaling questions of RoB 2, the main reason for a high risk of bias due to deviations from intended interventions in the five studies (1, 50–52, 54) was no blindness information for either experimenter or participants. Moreover, they did not require participants to have a diary and ensure adherence.

Additionally, one study (49) had a high-risk bias arising from the randomization, because the study did not report allocation concealment and had the baseline imbalance issue. Other 11 studies were rated as “unclear risk of bias” in this item, for there was no information about allocation concealment. Finally, only Filipas et al. (54) and Oaten and Cheng (47) showed outcome assessors were not aware of the training programme received. Others had some concerns about bias in the measurement of the outcome.

### Population Characteristics

Table 2 contains all information regarding relevant study characteristics. The total number of participants was 951. The male: female participant ratio was 56:125 (285 males and 666 females). The average age of the participants ranged from 18 (45) to 33.8 years (54). University students constituted the main population of the selected studies.

### Prior Mental Exertion Task

To examine the counteractive effect on mental fatigue, experimentally inducing mental exertion is necessary (58). However, different types and durations of mental exertion tasks were detected in the current study.

Specifically, two types of tasks were recruited by the previous studies to perform the mental exertion: emotional [e.g., anger-induced (48)] and cognitive tasks (other 11 studies presented in Table 2). Regarding the duration, the majority of the studies utilized 5 min to perform the mental exertion (19, 45–47, 50–52), while the longest duration was 90 min (54).

### Training Programme Characteristics

The majority of studies trained self-regulatory strength from cognitive domains. Mainly, they are posture regulation (19), studying programme (45), verbal mannerism modification (1), non-dominant hand use (1, 53), financial monitoring (47), Stroop and stop-signal task (49, 53), regular logical reasoning (50), and food-specific inhibition (52). Among all these training programmes related to cognitive domains, the longest training length is 4 months for the financial monitoring (47) and the shortest length is 1 week for regular logical reasoning (50). The majority of the included studies did not report the frequency of training, probably participants were expected to maintain the training at all times in their daily life. In contrast, studies that recruited some cognitive tasks showed this duration and frequency of training. They are Stroop task (3 times/day and 10 min/time) [(49), study 1], complex counting task (3 times/day and 10 min/time) [(49), study 2], and stop-single task (1 time/day) (52).

Moreover, some studies recruited physical training programmes as the intervention, such as cardiovascular exercise (46), isometric handgrip exercise (51), and the incremental maximal ramp with a cycle ergometer (54). Specifically, Bray et al. (51) used a 4-week and 3–4 times/week programme for cardiovascular exercise; Oaten and Cheng (46) recruited isometric handgrip exercise with 2 weeks and 2 times/day; and finally, Filipas et al. (54) used a 4-week incremental maximal ramp on cycle ergometers with 3 times/week and 60 min/time.

### Performance Outcomes

Outcomes including physical and cognitive performance are presented in Table 3. To operationally determine the effects that manipulation of self-regulatory strength has in improving physical and/or cognitive performance, the significant value in the experimental group compared with the control group was recruited.

**Table 3:** Summary of findings table for self-regulatory strength training programmes.

**Outcomes**	**Certainty assessment**					**Impact**	**No of participants and studies**	**Certainty of evidence (GRADE)**
	**Risk of Bias**	**Inconsistency**	**Indirectness**	**Imprecision**	**Other**			
Physical Performance assessed with: Handgrip Test follow-up: range 2to 6 weeks^a^	**Serious** ^**e**^	**Serious** ^**f**^	**Not serious**	**Serious** ^**g**^	**None**	Mixed findings among studies. Three studies showed improvements while one study did not find any change regarding persistence time in the handgrip test.	**305 (4 RCTS)**	⊕○○○ **VERY LOW**
Physical Performance assessed with: Cycling Ergometer follow-up: range 2 to 4 weeks ^b^	**Serious** ^**e**^	**Not serious**	**Not serious**	**Serious** ^**g**^	**None**	Participants exposed to the training programme experienced improvements in cycling performance.	**71 (2 RCTS)**	⊕⊕○○ **LOW**
Cognitive Performance assessed with: Inhibition follow-up: range 4 to 16 weeks ^c^	**Serious** ^**e**^	**Not serious**	**Not serious**	**Serious** ^**g**^	**None**	Participants exposed to the training programme experienced improvements in inhibition measured by Stroop Task, Visual Tracking Task.	**398 (8 RCTS)**	⊕⊕○○ **LOW**
Cognitive Performance assessed with: Problem-solving follow-up: range 1 to 2 weeks ^d^	**Serious** ^**e**^	**Not serious**	**Not serious**	**Serious** ^**g**^	**None**	Participants exposed to the training programme experienced improvements in problem-solving measured by Anagram Task.	**185 (3 RCTS)**	⊕⊕○○ **LOW**

*^a^Outcome including Muraven et al. (19); Cranwell et al. [(49): study 1 and study 2]; and Miles et al. (53)*.

*^b^Outcome including Bray et al. (51) and Filipas et al. (54)*.

*^3^Outcome including Oten and Cheng (45); Oaten and Cheng (46); Gailliot et al. [(1): study 4]; Oaten and Cheng (47); Denson et al. (48); Cranwell et la. [(49): study 1]; Allom and Mullan [(52): study 1 and study 2]*.

*^d^Outcome including Gailliot et al. [(1): study 1 and study 2]; Bertrams and Schmeichel (50)*.

*^e^Including study showed the high risk of bias. Therefore, the certainty of the evidence was downgraded*.

*^f^Including study showed inconsistent results with other studies. Therefore, the certainty of the evidence was downgraded*.

*^g^Including study did not use power analysis to determine sample size. Therefore, the certainty of the evidence was downgraded*.

*GRADE Working Group grades of evidence High certainty: we are very confident that the true effect lies close to that of the estimate of the effect. Moderate certainty: we are moderately confident in the effect estimate: the true effect is likely to be close to the estimate of the effect, but there is a possibility that it is substantially different. Low certainty: our confidence in the effect estimate is limited: the true effect may be substantially different from the estimate of the effect. Very low certainty: we have very little confidence in the effect estimate: the true effect is likely to be substantially different from the estimate of effect*.

#### Physical Performance Outcome

Four studies, including five investigations, showed the results of physical performance related to endurance performance in handgrip tasks (19, 49, 53) and ergometer cycling (54).

Specifically, according to the strength model of self-regulation of muscle analogy, Muraven et al. (19) first proposed that longitudinally repeated exercises of self-regulation could strengthen the resource (muscle). Muraven et al. (19) measured handgrip after 2-week posture and mood regulation, which required participants to maintain good posture (e.g., sit up straight and walk erectly) and mood all the time. The improvement index (19) (Table 3) showed a smaller decrease in persistent time of handgrip test after mental exertion in the posture-regulation group (+7.0) when compared to the mood-regulation group (−8.6). To find a more effective and accurate way to maintain compliance, Cranwell et al. (49) utilized a smartphone application to implement the training programme. Four weeks of Stroop (color identification) and counting (standing with one leg while counting backward from 1,000 in multiples of seven) task significantly improved persistence in handgrip compared to the control groups (study 1 Stroop training programme: *F*_(1, 25)_= 6.11, *p* < 0.02, $\eta_{p}^{2}$ = 0.196; study 2 counting training programme: *F*_(1, 30)_ = 15.09, *p* < 0.001, $\eta_{p}^{2}$ = 0.335). Thus, the muscle analogy was corroborated by the strength model of self-regulation. Based on these results, Filipas et al. (54) found that a 4-week cycle ergometer with incremental maximal ramp training could increase tolerance for mental exertion and improve total distance in endurance tests compared to the non-intervention group (*F*_(1, 19)_ = 5.66; *p* = 0.03).

To investigate more comprehensively from lab-based (handgrip) to real-life outcome (well-being), Miles et al. (53) recruited a 6-week training programme, including behavioral (non-dominant hand using for all daily activities) and cognitive (Stroop and stop-signal tasks) domains. More closely related to the current review concerning the outcome, the result inconsistently showed no significant difference in the persistent duration of the handgrip test between the training and control groups [*F*_(1, 171)_= 3.37, *p* = 0.07, $\eta_{p}^{2}$ = 0.02). Notably, Miles and colleagues detected a significant difference in well-being between the experimental and control groups; however, the significant value disappeared when controlling the covariates (trait self-control and conscientiousness) (*F*_(3, 167)_= 1.83, *p* = 0.14). Such effect is not elaborated on in the current review, because the result of well-being is out of the scope. However, future research should not disregard the covariates of physical and cognitive performance to better understand the effects of the a self-regulatory training programme.

#### Cognitive Performance Outcome

Eight studies examined the cognitive performance after training programmes. Specifically, to test whether self-regulation is operating like a “muscle,” Oaten and Cheng provided more “ecologically valid” contexts, including regular academic study, physical exercise, and financial monitoring of everyday self-regulatory behaviors. The results showed the three training programmes significantly decreased error: *F*_(1, 44)_ = 2,395.40, *p* < 0.001 (45), *F*_(1, 23)_= 966.34, *p* < 0.001 (46), and *F*_(1, 47)_ = 1,690.20, *p* < 0.001 (47) in visual tracking task, respectively. However, whether the effect of these training programmes could last for a certain period is not clear.

Bertrams and Schmeichel (50) first tested cognitive performance in anagram task after a week and recruited a training programme associated with the more complex cognitive process of logical reasoning. The results showed the number of anagrams solved in the experimental group increased considerably after the intervention compared to the control group [*F*_(1, 47)_ = 5.05, *p* = 0.03, $\eta_{p}^{2}$ = 0.11]. However, the performance did not improve at the follow-up test (after 1 week), which indicated the effect of the training programme might be temporary. The authors further argued that a variety of executive functions (e.g., logical reasoning) and self-regulation share one common resource, based on previous studies [e.g., (59, 60)]. A similar outcome in anagram was also obtained by Gailliot et al. (1) (study 1 and study 2) after 2 weeks of self-regulatory training programmes (verbal mannerism modifying and non-dominant hand using). However, motivation plays a key role. Specifically, the significant results were only detected in the low-motivation group, rather than the high-motivation group (1).

To investigate different dependent measurements, such as the Stroop task, Gailliot et al. (1) (study 4) found marginally higher accuracy in the experimental group (*M* = 97.50, *SD* = 3.04) compared to the control group (*M* = 94.04, *SD* = 6.81) after 2 weeks of self-regulatory training programme using non-dominant hands. Moreover, Cranwell et al. (49) (study 1) found that 4 weeks of smartphone application-based Stroop task training programme can significantly improve reaction time in the experimental group compared to the control group (*M* = 725.39, *SD* = 207.06 vs. *M* = 985.22, *SD* = 197.26, *F*_(1, 26)_ =10.84, *p* = 0.003, $\eta_{p}^{2}$ = 0.294). Furthermore, to conduct more ecologically valid research and determine whether increased self-regulation strength could transfer from a lab setting to the real world, Allom and Mullan (52) conducted two studies and found that after a certain period of self-regulatory strength training, the vulnerability of depletion among participants reduced dramatically, rather than health outcomes (e.g., eating behavior and body mass). More important to the current review, the cognitive outcomes showed inconsistent results. Specifically, when participants performed 20 number trials of the Stroop task, there was no significant difference in the reaction time (*p* > 0.05). In study 2, Allom and Mullan increased the number of trials in the Stroop task to 50, and the results showed a significant difference in the reaction time among food-specific (*M* = 32.10, *SD* = 69.64), general (*M* = 45.33, *SD* = 35.21), and no inhibition (*M* = 132.45, *SD* = 35.21) groups. However, consistent with the previous study (50), follow-up test after 1 week of training programme did not show any differences among food-specific (*M* = 108.92, *SD* = 74.55), general (*M* = 115.03, *SD* = 84.25), and no inhibition (*M* = 122.33, *SD* = 86.05) groups. Therefore, Allom and Mullan argued that a certain training paradigm could strengthen self-regulation, but does not necessarily benefit health behaviors (e.g., rejecting chocolate) in real life associated with self-regulatory strength. Moreover, improvements in self-regulation could not be maintained over time.

Finally, to test a different dependent measurement (impulsive aggression), Denson et al. (48) conducted a 2-week non-dominant hand training programme. The result showed that impulsive aggression was significantly reduced among the participants who were high in trait aggression (*t*_(66)_ = 2.15, *p* = 0.04) (48). The study further demonstrated that the effect of self-regulatory strength is cross-domain in various performances.

### Summary of Findings

The summary of findings table (Table 3) shows the certainty of evidence assessment based on different outcomes in the subsequent performance. Overall, the certainty or quality is low due to the serious risk of bias and imprecision. All included studies initially were graded from high-level certainty because their study designs are RCTs. Then, the overall certainty was downgraded, if necessary, as each domain was assessed. Finally, the evidence for physical performance measured by the handgrip test was downgraded to very low as a result of inconsistency (53), high risk of bias (49), and imprecision in sample size calculation (19, 49, 53) in the reported studies. Meanwhile, the other three outcomes (see Table 3) showed downgraded scores due to the high risk of bias (1, 50–52, 54) and imprecision (45–50, 54).

## Discussion

In this review, we sought to evaluate the literature on the manipulation of self-regulatory strength to counter mental exertion and improve physical and/or cognitive performance according to the strength model of self-regulation. The results shed light on the intervention for future studies.

### Prior Mental Exertion

The duration of the majority of prior mental exertion is less than 30 min (see Table 2), which has been recognized as a cut point of ego depletion and mental fatigue (33, 39). Only one study recruited a 90-min mental exertion (54) programme and investigated the intervention to counter mental fatigue. Since various durations of cognitive stimulation may have different effects (61, 62) of prior mental exertions on subsequent performance, it raises a variety of questions. For example, could a longer duration of prior mental exertion induce a higher level of fatigue? Can self-regulatory training programmes increase sufficient strength to counter all the exertions? And perhaps more likely, could the ensuing 2 weeks of strength training (typically used in previous studies; see Table 2) be of insufficient intensity and duration to have an impact on the effect of pre-mental exertion of more than 30 min in ‘mental fatigue' subject area [e.g., (34, 62, 63)] This may be the reason for the study of Filipas et al. (54) to recruit 4 weeks of the training programme and counter mental fatigue, rather than 2 weeks of training.

The majority of extracted studies recruited prior mental exertion, which tested inhibition, such as thought suppression (1, 19, 45–47), letter typing (50, 52), four consecutive tasks (53), and a Stroop task (49, 51). Inhibition refers to controlling one's impulse about attention, emotion, and behavior to override an automatic response (64, 65), which usually happens in sports scenarios. For example, soccer players should exert inhibition frequently to suppress an ongoing activity because they perform in a rapidly changing environment (66, 67) and easy to get mental fatigue in a prolonged duration of matches (90 min). Therefore, it may be promising to implement some training programmes in the subject areas of “ego depletion” (see Table 2) and “mental fatigue” to counter fatigue and improve subsequent performance in sports. However, future studies should consider the duration of the training programme.

Notably, the strength model indicates that self-regulation is a global resource account, suggesting a domain interaction for ego depletion, and a prominent analysis investigated by Hagger (9) first showed the same effects for matched and unmatched tasks.

The current review supported this “global” hypothesis and indicated training programmes could counter prior mental exertion tasks in different domains. For example, Denson et al. (48) recruited a non-dominant hand using programme to increase self-regulatory strength. The result showed the counteractive effect could appear in the emotional domain with the Taylor aggression paradigm. Thus, future studies could consider manipulating training programmes to counter prior mental exertion in different domains, such as emotion and cognition.

More to the point, the study (48) shed light on the fact that emotional regulation could consume the same resource pool of self-regulation and can also be increased by the self-regulatory strength training. According to the neurovisceral integration model (68, 69), emotional, cognitive, and behavioral self-regulation is correlated with the autonomic nervous system measured by some physiological indicators, such as heart rate variability (70, 71) and skin conductance response (72, 73). Also, because these indicators changed significantly when an individual is involved in social interactions (74, 75), these training programmes may be beneficial for some behaviors, such as the maintenance of comfortable interpersonal space and defensive responses of fearful faces. However, this hypothesis should be tested by future studies.

### Training Programmes

A variety of training programmes were investigated. Among them, non-dominant hand use is eye-catching, as it was used in four investigations [(1, 48), study 2 and study 4 (53)]. Motor movements with the non-dominant hand are less intuitive and spontaneous, necessitating the use of greater cognitive resources (self-regulation) (76). Image studies have verified that using the non-dominant hand interferes with cognitive processing, and executing a motor task with the non-dominant hand increases cortical activity (77). Consistently, According to Jäncke et al. (78), executing a consecutive movement with the non-dominant hand (in right-handed subjects) leads to increased right hemisphere activation. Thus, it is not surprising that completion number in anagram and accuracy in Stroop task were increased, respectively [(1) study 2 and study 4], because the strength of self-regulation was improved after a 2-week training programme. However, this increased *strength* seems to be temporary after training programmes, and two studies did not find significant improvement at the follow-up test (1 week after the test) (50, 52).

In the recent decade, the resource model has met many challenges. For example, many scholars questioned what is exactly the resource if self-regulation depends on a limited resource? Gailliot et al. (1) conducted a study to manifest that the metaphorical resource may be blood glucose. Nevertheless, this hypothesis was never tested successfully despite its compelling attractiveness (79). Finally, Finley et al. (80) conducted the most stringent test and found no evidence to support the glucose hypothesis. Although the current review supports the resource model and showed the strength of self-regulation can be improved, future studies should examine the mechanism that underlies this improvement.

Moreover, motivation was placed at the center of self-regulation, rather than a resource, such as a process self-regulation model (81, 82). Specifically, these researchers questioned if individuals are unable to restrain themselves due to a lack of resources, observing how motivating incentives might instantaneously reverse depletion. For example, some studies showed providing additional motivation (e.g., being kind to participants or telling participants the research would have a good cause) could ease the effect of ego depletion (83, 84). Also, the effect of ego depletion could be buffered by either the idea of money (85) or real money (86), because people are motivated to conserve more resources for the next stage (20).

The resource model has been extensively updated. For example, Baumeister and Vohs (13, 20) acknowledged the deficiency and remedied motivation as an ingredient/variable in the model. That is, *motivation* plays a role in the mobilization of *strength*. The authors argued that if people have high motivation and want to measure up to some certain standard, this may compensate for somewhat low self-regulatory resources or enhance the monitoring process. Motivational differences may cause inconsistent results between the study of Miles et al. (53) and other studies listed in Table 2.

Therefore, the current review argued that the strength of the self-regulatory training programme makes humans less vulnerable to ego depletion or mental fatigue; however, the effect of these training programmes is dependent on motivation.

### Performance Outcome

To evaluate the performance outcome, the current review follows the study of Friedman and Miyake (87) by recognizing the suggested categorization on which the matching of depleting and dependent programs was based. For example, all performance outcomes related to impulse control tasks (e.g., Stroop task, handgrip, and aggression inhibition) are categorized as “inhibition” (see Table 2: Domain of the Outcome).

From this categorization, the current review found the effect of these training programmes could be cross-domain, since many studies recruited unmatched types of tasks (Table: Similarity) between intervention and performance and showed significant improvement [(49, 51), study 2 (1), study 1 and 2 (46)]. This finding supports the strength model of self-regulation that when training this *strength* in a domain, it can improve the performance in an unrelated task (11, 13, 17, 20). Thus, it confirms the findings described in Section Prior Mental Exertion, which shows the resource of self-regulation is “global”.

It is worthy to further note that Gailliot et al. (1) (study 1 and study 2) used the lower-level executive function of inhibition to increase self-regulatory strength, while the higher-level executive function of problem-solving improved considerably. Therefore, the cross-domain improvement scenario can occur at different orders of executive function (low vs. high order) [see Diamond (64): the clarification of executive functions].

Based on this review, only one investigation examined the training programme to overcome mental fatigue and improve subsequent endurance performance (54). Here, we highlight the caution, because this study did not explicitly mention that the training programme is related to the strength of self-regulation. Nevertheless, we include it because numerous studies have shown that regular physical exercise can increase self-regulation resources (88, 89), even though it did not report or measure the ability of self-regulation.

## Limitations

This systematic review poses a few noteworthy limitations. First, this systematic review, conducted rigorously, is not a meta-analysis, because of the heterogeneity across measurement and training programmes. Moreover, the suggested categorization of self-regulatory tasks (between intervention and performance test) did not present specific task demands, such as inhibition vs. initiating actions. Thus, a future study can investigate more details about this similarity between the training programme and performance test to see the cross-domain effect of self-regulatory strength. Finally, selecting only publications written in English may further limit the representation of the results.

## Conclusion

Overall, *strength* as an important ingredient in the resource model can be trained to counter prior mental exertion and improve subsequent cognitive and physical performance. Cross-domain effects (emotional and cognitive domains; higher and lower levels of executive functions) were found for self-regulatory strength. However, *motivation* plays a key role to mobilize this resource. Future studies should examine the mechanism that underlies the *strength* and should also apply these training programmes for social interactions.

## Data Availability Statement

The original contributions presented in the study are included in the article/Supplementary Material, further inquiries can be directed to the corresponding author/s.

## Author Contributions

All authors listed have made a substantial, direct, and intellectual contribution to the work and approved it for publication.

## Conflict of Interest

The authors declare that the research was conducted in the absence of any commercial or financial relationships that could be construed as a potential conflict of interest.

## Publisher's Note

All claims expressed in this article are solely those of the authors and do not necessarily represent those of their affiliated organizations, or those of the publisher, the editors and the reviewers. Any product that may be evaluated in this article, or claim that may be made by its manufacturer, is not guaranteed or endorsed by the publisher.
