# Supplementary material for: Can Self-Regulatory Strength Training Counter Prior Mental Exertion? A Systematic Review of Randomized Controlled Trials
Source: Front Public Health. 2022 Jun 10;10:904374. doi: 10.3389/fpubh.2022.904374 (PMC9226420; doi:10.3389/fpubh.2022.904374)
Supplement: Supplementary file 1 [file Table_1.DOCX]

**Table S1 – Details of searching strategy**

| **Database** | **Search Keywords** | **Results** |
| --- | --- | --- |
| Pubmed | 1. **(((willpower) OR (inhibit*)) OR (self-control)) OR (self-regulat*) 3,128,230** 2. **((((((interven*) OR (attenuate)) OR (counter*)) OR (improv*)) OR (exercis*)) OR (practice*)) OR (training), 7,005,505** 3. **(((ego depletion) OR (cognitive fatigue)) OR (mental exertion)) OR (mental fatigue), 20,497** 4. (1) AND (2) AND (3), **1,335** 5. (3) limits published date 1999 - Jan 2022, **1,267** 6. (4) limits randomized controlled trial, **301** | 301 |
| Web of Science | 1. TS=(willpower OR inhibit* OR self-control OR self-regulat*), **4,596,897** 2. TS=(interven* OR attenuate OR counter* OR improv* OR exercis* OR practice* OR training), **12,137,055** 3. TS=("ego depletion" OR "mental fatigue" OR "mental exertion" OR "cognitive fatigue"), **6,148** 4. (1) AND (2) AND (3), **663** 5. (3) limits published date: 1999 – Jan 2022, **651** 6. (3) limits English, **639** | 639 |
| EBSCOhost | 1. (willpower OR inhibit* OR self-control OR self-regulat*) AND (interven* OR attenuate OR counter* OR improv* OR   exercis* OR practice* OR training) AND ("ego depletion" OR "mental fatigue" OR "mental exertion" OR "cognitive fatigue"), **2,192**   1. (1) limits published date: 1999 – Jan 2022, **2,157** 2. (2) excludes Magazines, books and Reviews, **2,109** 3. (3) excludes duplicates in EBSCOhost folder, **1797** | 1,797 |
| Scopus | 1. TITLE-ABS-KEY ( ( willpower  OR  inhibit*  OR  self-control  OR  self-regulat* )  AND  ( interven*  OR  attenuate  OR  counter*  OR  improv*  OR  exercis*  OR  practice*  OR  training ) AND  ( "ego depletion"  OR  "mental fatigue"  OR  "mental exertion"  OR  "cognitive fatigue" ) ), **289** 2. (1) limits published date: 1999 – Jan 2022, **282** 3. (2) excludes Review, **257** | 257 |
